# Reaction of benzoxasilocines with aromatic aldehydes: Synthesis of homopterocarpans

**DOI:** 10.1186/1860-5397-3-5

**Published:** 2007-02-08

**Authors:** Míriam Álvarez-Corral, Cristóbal López-Sánchez, Leticia Jiménez-González, Antonio Rosales, Manuel Muñoz-Dorado, Ignacio Rodríguez-García

**Affiliations:** 1 Dpto. Química Orgánica, Universidad de Almería, 04120-Almería, Spain

## Abstract

Condensation of 2*H*-benzo[*g*][1,2]oxasilocines with aromatic aldehydes in the presence of boron trifluoride affords mixtures of *cis/trans* 2-phenyl-3-vinylchromans with moderate yields. These can be transformed into homopterocarpans, a synthetic group of substances homologous to the natural isoflavonoid pterocarpans.

## Background

**Scheme 1:**
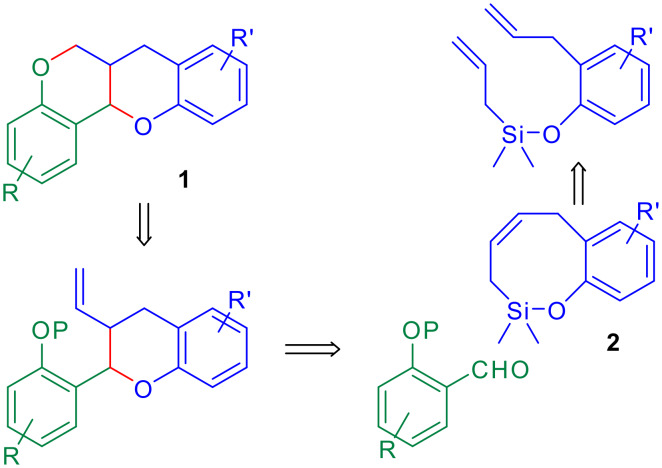
Retrosynthetic analysis for the homopterocarpan skeleton.

The Sakurai-Hosomi is a useful variant of allylation reactions, [1] which has been used for the formation of carbo- and heterocycles. [2–3] We have applied it to the stereoselective synthesis of dihydrobenzofurans by means of the condensation of benzoxasilepines with aromatic aldehydes in the presence of Lewis acids. [4–5] Using this methodology and through convergent synthetic routes, we have prepared pterocarpans [6] and neolignans. [7] These good results have encouraged us to undertake the extension of the method to the use of benzo [*g*][1,2]oxasilocines for the preparation of chromans. This heterocyclic system constitutes the core skeleton of several biologically active natural products [8–11] and it is also present in the basic structure of the homopterocarpans. [12] These are a group of non natural substances whose total synthesis [13] has been stimulated by their interesting biological activities, like antitumor [14] or potential anti-HIV. [15] A theoretical study of their structure has also been published. [16] Here we describe a concise and convergent approach to this skeleton (**1**) based on a Sakurai condensation between a benzoxasilocine (**2**) and a protected *ortho*-hydroxybenzaldehyde (Scheme 1).

## Results and discussion

### Starting materials

**Scheme 2:**
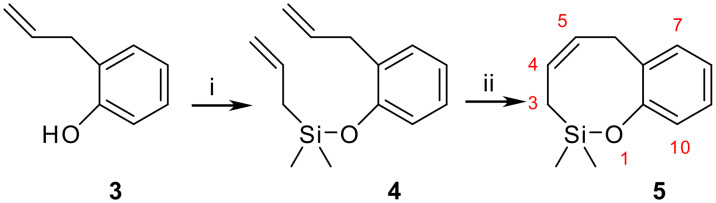
*Reagents*: i CH_2_ = CHCH_2_SiMe_2_Cl, Et_3_N, DCM, 85%; ii 2^nd^ generation Grubbs catalyst, DCM, 91%.

The starting material required for this synthesis is the novel heterocycle 3,6-dihydro-2,2-dimethyl-2*H*-benzo [*g*][1,2]oxasilocine (**5**), which can be prepared through ring closing metathesis (RCM), as has been previously reported for the non-benzofused system. [17–19] Thus, silylation of 2-allylphenol (**3**) (or conveniently functionalized derivatives) with allylchlorodimethylsilane followed by RCM with 2^nd^ generation Grubbs catalyst [20] leads to the cyclic siloxane with high yields (Scheme 2). The good results in the cyclization step make this approach an excellent way of synthesising of this heterocycle.

### Reaction of benzoxasilocines with aromatic aldehydes in the presence of BF_3_·Et_2_O

**Scheme 3:**
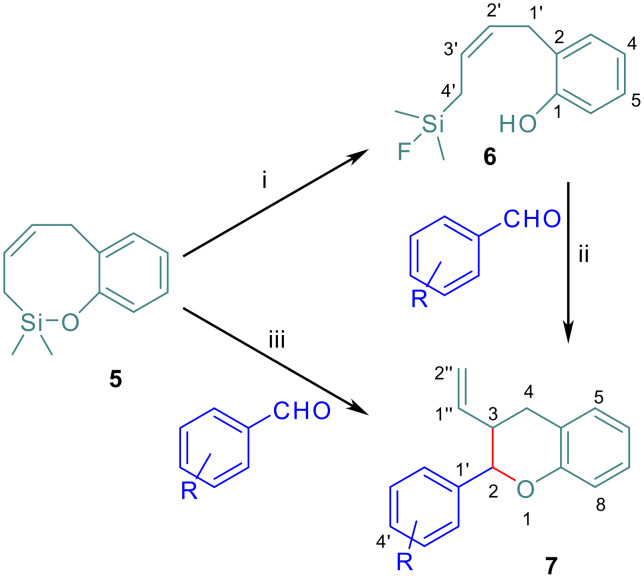
*Reagents*: i: BF_3_·Et_2_O (1 eq), MeOH 95%; ii: substituted benzaldehydes, BF_3_·Et_2_O (1 eq), DCM; iii: substituted benzaldehydes, BF_3_·Et_2_O (2 eq), DCM; see Table 1 for *cis/trans* ratios and yields.

We had previously observed [4] that the treatment of the seven membered cyclic allylsiloxane 2,3-dihydro-2,2-dimethylbenzo[*f*][1,2]oxasilepine with boron trifluoride yielded a ring-opened fluorinated derivative. This derivative was able to perform the condensation with aromatic aldehydes to generate the dihydrobenzofuran final products in the presence of a second equivalent of BF_3_·Et_2_O. In a similar way, when **5** is treated with BF_3_·Et_2_O in MeOH, the fluorinated species **6** is formed quantitatively (Scheme 3). The ^1^H NMR is very similar to that of the starting material, but for the methyl groups on silicon, which appear now as doublets due to their coupling with the ^19^F (^3^*J*_H-F_ = 7.3 Hz). This coupling is also observed for the methylene on silicon H4', which exhibits now an additional splitting (^3^*J*_H-F_ = 6.5 Hz) (for details see Supporting Information File 1). ^13^C NMR also reveals the presence of the fluorine on the silicon, because the signal due to the methyl groups appears as a doublet (^2^*J*_C-F_ = 14.8 Hz) as well as the signal due to C4' (^2^*J*_C-F_ = 13.5 Hz). ^19^F NMR shows only one signal at -160.73 ppm (hept t, ^3^*J*_F-H_ = 7.3 Hz, ^3^*J*_F-H_ = 6.5 Hz) with satellite bands due to the ^19^F-^29^Si coupling (^2^*J*_F-Si_ = 283 Hz). A similar spectroscopic behaviour has been reported for other fluorosilanes. [4,21]

**Table 1:** Condensation of substituted benzaldehydes with **5**.

benzaldehyde substituent	product	diastereomeric ratio (*cis/trans*)^a^	yield (DCM)^b^	yield (CHCl_3_)^c^

H	**7a**	1 : 3	49	56
2-OMe	**7b**	1 : 1	51	58
3-OMe	**7c**	1 : 3	30	36
4-OMe	**7d**	1 : 5	48	60
2-OPiv	**7e**	1 : 3	48	62
3-OPiv	**7f**	1 : 5	42	44
4-OPiv	**7g**	1 : 3	47	58

^a^: As deduced by analysis of ^1^H NMR spectra or after CC separation ^b^: reflux; ^c^: 20°C.

In order to study whether the electronic nature of the aldehyde had any influence on the diastereochemical outcome of the reaction, as observed before with the benzoxasilepines, [4] a selection of benzaldehydes with strongly (OMe) or weakly (OPiv) electron donating groups in *ortho, meta* and *para* positions were assayed (Table 1). Under the same experimental conditions used for the preparation of dihydrobenzofurans, the reaction is never diastereospecific, as *cis/trans* mixtures are always observed, the *trans* isomer being the major one. In addition, no clear influence of the electron density of the carbonyl on the diastereomeric ratio can be established. The yields are also considerably lower than those for the dehomologous system. The lack of conjugation between the allylsiloxane double bond in **5** or in **6** when compared with the analogous seven-membered benzoxasilepine could enhance the reactivity and instability of these compounds, accelerating the reaction but also increasing its rate of decomposition. When the reaction is performed in CHCl_3_, a slight increase in the yields is observed, but the diastereoselection levels are basically the same.

We have also described that benzoxasilepines can be condensed with benzaldehydes in the presence of a stoichiometric amount of KF and 18-crown-6 and a catalytic amount of a complex formed with AgOTf and (±)-BINAP to give good yields of dihydrobenzofurans. [5] Under the same reaction conditions, the eight membered benzoxasilocines did not react.

The *cis/trans* diastereoisomers could be easily distinguished by means of the coupling constants between the protons H2 and H3 in ^1^H NMR, which range from 1.6 Hz to 3.5 Hz for the *cis* isomers and 8.4 Hz to 9.5 Hz for the *trans*.

**Scheme 4:**
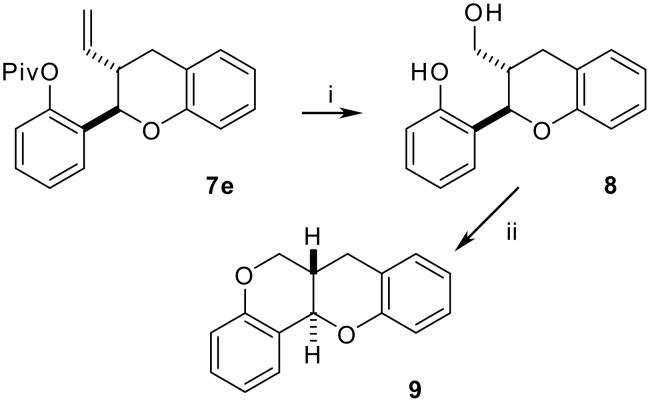
*Reagents*: i: a) OsO_4_, KIO_4_, THF-H_2_O, 79%; b) LiAlH_4_, Et_2_, 0°C, 76%; iii: PPh_3_, DIAD, THF, 70%.

Compound **7e** was used for the preparation of the core skeleton of homopterocarpan (Scheme 4). Degradation of the olefinic double bond with OsO_4_/KIO_4_ afforded an aldehyde which was reduced with LiAlH_4_. Under these conditions the pivaloyl protecting group was removed, affording the dihydroxylated derivative **8**. Application of the Mitsunobu conditions (DIAD, PPh_3_) to **9** promoted the cyclization to give the homopterocarpan **8**.

Therefore, following this five steps route, we have accessed the skeleton of homopterocarpan in a convergent approach. We plan to use this strategy for the preparation of a variety of derivatives conveniently substituted on both aromatic rings through an appropriate selection of the starting benzoxasilocine and aromatic aldehyde. In addition, access to the *cis* isomers would allow the study of structure-activity relationships when compared with the *trans* isomers.

## Conclusion

The condensation of benzoxasilocines with aromatic aldehydes in the presence of boron trifluoride has been studied. Yields are lower than those for the benzoxasilepines, and the diastereoselectivity is not directly influenced by the electronic density of the aldehydes. Mixtures of *cis/trans* 2-phenyl-3-vinylchromans are always formed, but the *trans* isomer dominates.

It is also described a new total synthesis of homopterocarpan skeleton, which is based on an appropriate transformation of the *trans*-2-(2-pivaloyloxyphenyl)-3-vinylchroman prepared through Sakurai reaction. In this way we have outlined an alternative synthetic strategy for the preparation of non natural analogs of the pterocarpans with promising biologic activities.

## Supporting Information

File 1 Experimental data. This file contains all experimental methods and analytical data belonging to the compounds described in the article.

## References

[1] Hosomi A, Sakurai H (1976). Tetrahedron Lett.

[2] Chabaud L, James P, Landais Y (2004). Eur J Org Chem.

[3] Sarkar T K, Haque S A, Basak A (2004). Angew Chem, Int Ed Engl.

[4] Jiménez-González L, García-Muñoz S, Álvarez-Corral M, Muñoz-Dorado M, Rodríguez-García I (2007). Chem–Eur J.

[5] Jiménez-González L, Álvarez-Corral M, Muñoz-Dorado M, Rodríguez-García I (2006). Chem–Eur J.

[6] Jiménez-González L, Álvarez-Corral M, Muñoz-Dorado M, Rodríguez-García I (2005). Chem Commun.

[7] García-Muñoz S, Jiménez-González L, Álvarez-Corral M, Muñoz-Dorado M, Rodríguez-García I (2005). Synlett.

[8] Decosterd L A, Evans H S, Msonthi J D, Hostettmann K (1987). Helv Chim Acta.

[9] Furusawa M, Ido Y, Tanaka T, Ito T, Nakaya K, Ibrahim I, Ohyama M, Iinuma M, Shirataka Y, Takahashi Y (2005). Helv Chim Acta.

[10] Nkunya M H H, Waibel R, Achenbach H (1993). Phytochemistry.

[11] Tanaka T, Ito T, Iinuma M, Takahashi Y, Naganawa H (1998). Phytochemistry.

[12] Valenti P, Montanari P, Barili P L, Dare P (1980). Arch Pharm.

[13] Da Re P, Valenti P, Cateni L, Barili P L (1976). Tetrahedron Lett.

[14] Rampa A, Bisi A, Belluti F, Gobbi S, Piazzi L, Valenti P, Zampiron A, Caputo A, Varani K, Borea P A (2005). Farmaco.

[15] Valenti P, Fabbri G, Rampa A, Bisi A, Belluti F (1997). Farmaco.

[16] Schoning A, Friedrichsen W (1989). Z Naturforsch, B: Chem Sci.

[17] Chang S B, Grubbs R H (1997). Tetrahedron Lett.

[18] Meyer C, Cossy J (1997). Tetrahedron Lett.

[19] Yao Q W (2000). Angew Chem, Int Ed Engl.

[20] Scholl M, Ding S, Lee C W, Grubbs R H (1999). Org Lett.

[21] Knolker H J, Wanzl G (1995). Synlett.

