# Supplementary material for: Reaction of benzoxasilocines with aromatic aldehydes: Synthesis of homopterocarpans
Source: Beilstein J Org Chem. 2007 Feb 8;3:5. doi: 10.1186/1860-5397-3-5 (PMC1810298; doi:10.1186/1860-5397-3-5)
Supplement: File 1 — Experimental data. This file contains all experimental methods and analytical data belonging to the compounds described in the article. [file Beilstein_J_Org_Chem-03-05-s001.doc]

**Reaction of benzoxasilocines with aromatic aldehydes.**

**Synthesis of homopterocarpans**

Míriam Álvarez-Corral, Cristóbal López-Sánchez, Leticia Jiménez-González, Antonio Rosales, Manuel Muñoz-Dorado and Ignacio Rodríguez-García*

**Additional material:**

**Experimental**

**General**

Infrared spectra were recorded in liquid film between NaCl plates on a FT-IR Mattson Genesis II. NMR spectra were determined on a Bruker Avance DPX 300 and Bruker Avance-500. ^1^H NMR and ^13^C NMR spectra were recorded in deuterated solvent and are reported to tetramethylsilane. Carbon substitution degrees were established by DEPT multipulse sequence, and ^13^C NMR peak assignments were made with the aid of 2D NMR (HMBC, HMQC, COSY and NOESY). HRMS were registered on an Autospec-Q VG Analytical (FISONS) mass spectrometer. All solvents were purified and dried following standard procedures[[1]](#footnote-2).

**Allyl-(2-allylphenoxy)-dimethylsilane (4)**

Commercially available 2-allylphenol (**3**) (2g, 14.9mmol) was dissolved in anhydrous CH_2_Cl_2_ (75 mL) at 0ºC under N_2_ atmosphere. Anhydrous NEt_3_ (2.5 mL, 17.9 mmol) and allylchlorodimethylsilane (2.4 mL, 16.4 mmol) were added. The reaction mixture was stirred at 0ºC for 3h, and then, a saturated solution of NaHCO_3_/H_2_O was added The whole mixture was extracted with CH_2_Cl_2_ (3 x 50 mL). The organic layer was dried over anhyd. Na_2_SO_4_, and concentrated *in vacuo*. Flash chromatography (hexane: Et_2_O, 9:1) of the residue gave **4** (2.91 g, 12.5 mmol, 85%) as a colourless oil: IR (film) ν_max_ 3076, 2961, 2903, 1631, 1557, 1489, 1451, 1253, 1159, 1040, 920, 834, 756. ^1^H NMR (CDCl_3_, 300 MHz) δ 7.17 (2H, m, H-3, H-5), 6.97 (1H, dt, *J*= 7.2, *J*=1.2 Hz, H-4), 6.85 (1H, dd, *J*=6.9, *J*=1.2 Hz, H-6), 5.94 (2H, m, H-2’,2’’), 5.00 (4H, m, H-3’, H-3’’), 3.40 (2H, d, *J*= 6.9 Hz, H-1’), 1.84 (2H, d, *J*=8.1 Hz, H-1’’), 0.35 (6H, s, -SiCH_3_). ^13^C NMR (CDCl_3_, 75 MHz) δ 153.00 (C, C-1), 136.97 (CH, C-2’), 133.23 (CH, C-2’’), 130.73 (C, C-6), 130.15 (CH, C-2), 127.08 (CH, C-3), 121.44 (CH, C-4), 118.71 (CH, C-5), 115.41 ( CH_2_, C-3’), 114.37 (CH_2_, C-3’’), 34.52 (CH_2_, C-1’), 24.78 (CH_2_, C-1’’), -1.59 (2CH_3_, Si(CH_3_)_2_). HREIMS (*m/z*) calcd. for C_14_H_20_OSi 232.1283 [M]^+^, found 232.1285.

**3,6-Dihydro-2,2-dimethyl-2H-benzo[*g*][1,2]oxasilocine (5)**

[1,3-Bis-(2,4,6-trimethylphenyl)-2-imidazolidinylidene)dichloro(phenylmethylene)-(tricyclohexylphosphine)ruthenium] (2^nd^ generation Grubbs catalyst) (54 mg, 0.06 mmol) was added to a stirred solution 0.02 M of **4** (500 mg, 2.15 mmol) in anhyd. CH_2_Cl_2_, under N_2_ atmosphere. The mixture was stirred under reflux for 3 h, and then, the solvent was removed *in vacuo*. Flash chromatography (hexane: Et_2_O, 98:2) of the residue gave **5** (400 mg, 1.96 mmol, 91%) as a colourless oil: IR (film) ν_max_ 2941, 2924, 2871, 2794, 1580, 1484, 1449, 1253, 1035, 925, 842, 800, 751.^1^H NMR (CDCl_3_, 300 MHz) δ 7.14 (2H, m, H-7, H-9), 6.95 (1H, dt, *J*=1.2, *J*= 7.3 Hz, H-8), 6.87 (1H, dd, *J*=1.2, *J*=6.5 Hz, H-10), 5.60 (2H, m, H-4, H-5), 3.36 (2H, d, *J*= 7.7 Hz, H-6), 1.85 (2H, d, *J*=7.3 Hz, H-3), 0.23 (6H, s, Si(CH_3_)_3_). ^13^C NMR (CDCl_3_, 75 MHz) δ 152.75 (C, C-10a), 134.45 (C, C-6a), 129.69 (CH,C-9), 128.91 (CH, C-7), 127.19 (CH, C-8), 123.96 (CH, C-10), 122.09 (CH, C-5), 121.35 (CH, C-4), 29.95 (CH_2_, C-6), 19.23 (CH_2_, C-3), -1.86 (2CH_3_, -Si(CH_3_)_2_). HREIMS (*m/z*) calcd. for C_12_H_16_OSi 204.0970 [M]^+^, found 204.0968.

**(*Z*)-2-(4-Fluorodimethylsilyl-2-butenyl)phenol (6).**

To a solution of **5** (50 mg, 0.24 mmol) in methanol (0.5 mL) BF_3_·Et_2_O (62 μL, 0.49 mmol) was added. The mixture was stirred at room temperature for 5 minutes. After that, the reaction was diluted with CH_2_Cl_2_ (10 mL) and washed with brine (5 mL). The organic phase was dried over MgSO_4_ and evaporated under vacuo to give compound **6** (49mg, 0.22 mmol) in a 95% yield as a colourless oil: IR (film) ν_max_ 3567, 3528, 3423, 2958, 2926, 2870, 1601, 1579, 1484, 1449, 1381, 1337, 1256, 1212, 1158, 1090, 874, 843, 798 cm^-1^. ^1^H NMR (300 MHz, CDCl_3_): 7.14 (1H, dd, *J*= 8.1, *J*= 7.3 Hz, H-5), 7.13 (1H, d, *J* = 7.3 Hz, H-3), 6.90 (1H, t, *J*= 7.3 Hz, H-4), 6.82 (1H, d, *J*= 8.1 Hz, H-6), 5.63 (2H, m, H-2’, H-3’), 5.06 (1H, bs, OH), 3.41 (2H, d, *J*= 6.5 Hz, H-1’), 1.86 (2H, dd, *J* = 7.3, ^3^*J*H-F = 6.5 Hz, H-4´), 0.30 (6H, d, ^3^*J*H-F = 7.3 Hz, (CH_3_)_2_SiF). ^19^FNMR (289 MHz, CDCl_3_): -160.73 (hept t, ^3^*J*F-H = 7.3 Hz, ^3^*J*F-H = 6.5 Hz). ^13^C NMR (75 MHz, CDCl_3_): 154.02 (C, C-1), 129.98* (CH, C-3), 127.55* (CH, C-5), 126.66 (CH, C-3´), 126.37 (C, C-2), 125.04 (CH, C-2’), 120.79 (CH, C-4), 115.63 (CH, C-6), 28.35 (CH_2_, C-1’), 18.50 (CH_2_, d, ^2^*J*C-F = 13.5 Hz, C-4´), -1.54 (CH_3_, d, ^2^*J*C-F = 14.8 Hz, C-4´). (*may be interchanged). HREIMS (*m/z*) calcd. for C_12_H_17_OSiF 224,1033 [M]^+^, found 224.1030.

**General procedure for the Sakurai reaction in DCM:** To a stirred solution of the corresponding benzoxasilocine in anhyd. CH_2_Cl_2_ (4 mL/mmol) under N_2_ atmosphere at ‑45 ºC, BF_3_·Et_2_O (2 equiv.) was added. After 5 min, a solution of the aldehyde (1.1 equiv.) in CH_2_Cl_2_ was added dropwise. The reaction mixture was stirred at –45 ºC for 2 h. and was allowed to warm to room temperature; afterwards was heated to reflux for 4-10 h., then diluted with CH_2_Cl_2_ and washed with brine. The dried (Na_2_SO_4_) extract was concentrated *in vacuo* and the *cis/trans* relative ratio was measured from ^1^H NMR. The major isomers were purified by chromatography over silica gel.

**General procedure for the Sakurai reaction in CHCl_3_:** To a stirred solution of the corresponding benzoxasilocine in anhyd. CHCl_3_ (7 mL/mmol) under N_2_ atmosphere at ‑45 ºC, BF_3_·Et_2_O (2 equiv.) was added. After 5 min, a solution of the aldehyde (1.1 equiv.) in CHCl_3_ was added dropwise. The reaction mixture was stirred at room temperature for 24h., then diluted with CH_2_Cl_2_ and washed with brine. The dried (Na_2_SO_4_) extract was concentrated *in vacuo* and the *cis/trans* relative ratio was measured from ^1^H NMR. The major isomers were purified by chromatography over silica gel.

***cis* and *trans* 2-phenyl-3-vinyl-2,3-dihydrobenzopyran (7a).**

Reaction of benzoxasilocine (**5**) (132mg, 0.65mmol) with benzaldehyde (77 mg, 0.73 mmol) and BF_3_·Et_2_O (187 mg, 1.32 mmol) in anhydrous CH_2_Cl_2_ (2.5 mL) followed by workup as described above yielded **7a** as a *cis/trans* mixture in a 1:3 relative ratio, with an overall yield of 49% (hexane/Et_2_O, 95:5); IR (film) ν_max_: 3066, 3029, 2954, 2854, 1615, 1585, 1488, 1456, 1307, 1240, 1109, 1015, 919, 803, 751. ^1^H NMR (CDCl_3_, 300 MHz) *(signals of cis isomer)*: δ 5.72 (1H, ddd, *J*=8.1, *J*=10.9, *J=* 18.6 Hz, H-1’’), 5.28 (1H, d, *J*=2.7 Hz, H-2), 4.92 (1H, d, *J*=18.6 Hz, H-2’’b), *(signals of trans isomer)*: δ 5.61 (1H, ddd, *J*=6.9, *J*=10.5, *J=* 17.3 Hz, H-1’’), 5.03 (1H, d, *J*= 18.1 Hz, H-2’’a), 5.00 (1H, d, *J*= 9.7 Hz, H-2’’b), 4.82 (1H, d, *J*=8.9 Hz, H-2), *(signals of both)*: δ 7.38 (m, H-2’-6’), 7.18 (m, H-5, H-7), 6.90 (m, H-6, H-8), 2.92 (3H, m, H-3, H-4). ^13^C NMR (CDCl_3_, 75 MHz): *(signals of trans isomer)* δ 154.64 (C, C-8a), 139.89 (C, C-1’), 137.52 (CH, C-1’’), 129.34 (CH, C-5), 128.34 (2CH, C-3’, C-5’), 128.12 (CH, C-4’), 127.39 (CH, C-7), 127.24 (2CH, C2’, C-6’), 121.27 (C, C-4a), 120.41 (CH, C-6), 116.52 (CH, C-8), 116.36 (CH_2_, C-2’’), 81.99 (CH, C-2), 42.44 (CH, C-3), 30.83 (CH_2_, C-4). HREIMS (*m/z*) calcd. for C_17_H_16_O 236.1201 [M]^+^, found 236.1204.

***cis* and *trans* 2-(2-methoxyphenyl)-3-vinyl-2,3-dihydrobenzopyran** **(7b).**

Reaction of benzoxasilocine (**5**) (55 mg, 0.27 mmol) with 2-methoxybenzaldehyde (41 mg, 0.32 mmol) and BF_3_·Et_2_O (68 μL, 0.54 mmol) in anhydrous CH_2_Cl_2_ (2.5 mL) followed by workup as described above yielded **7b** as a *cis/trans* mixture in a 1:1 relative ratio, with an overall yield of 51%. (hexane/Et_2_O, 95:5); IR (film) ν_max_: 2952, 2923, 2852, 1620, 1584, 1489, 1459, 1375, 1241, 1105, 1030, 916, 802, 751. ^1^H NMR (CDCl_3_, 300 MHz) *(signals of cis isomer)* δ 7.4 (1H, dd, *J*=1.9, *J*=7.7 Hz, H-6’), 5.78 (1H, ddd, *J*=7.8, *J*= 10.8, *J*=18.1 Hz, H-1’’), 5.56 (1H, d, *J*=2.4 Hz, H-2),4.97 (1H, m, H-2a”), 4.80 (1H, dt, *J*=17.4, *J*= 1.4 Hz, H-2b’’), 3.88 (3H, s, OMe), 3.37 (1H, dd, *J*=16.4, *J*=5.8 Hz, H-4a), 3.04 (1H, m, H-3), 2.89 (1H, m, H-4b), *(signals of trans isomer)* δ 7.53 (1H, dd, *J*=1.4, *J*=7.7 Hz, H-6’), 5.69 (1H, ddd, *J*=7.7, *J*= 11.2, *J*=18.1 Hz, H-1’’), 5.40 (1H, d, *J*=8.4 Hz, H-2), 4.97 (2H, m, H-2”), 3.84 (3H, s, OMe), 2.85 (3H, m, H-3, H-4), *(signals of both)* δ 7.32 (1H, m, H-3’), 7.15 (2H, m, H-5, H-7), 7.01 (1H, m, H-4’), 6.92 (3H, m, H-5’, H-6, H-8). HREIMS (*m/z*) calcd. for C_18_H_18_O_2_ 266.1307 [M]^+^, found 266.1309.

***cis* and *trans* 2-(3-methoxyphenyl)-3-vinyl-2,3-dihydrobenzopyran** **(7c).**

Reaction of benzoxasilocine (**5**) (85 mg, 0.42 mmol) with 3-methoxybenzaldehyde (63 mg, 0.47 mmol) and BF_3_·Et_2_O (106 μL, 0.84 mmol) in anhydrous CH_2_Cl_2_ (2.5 mL) followed by workup as described above yielded **7c** as a *cis/trans* mixture in a 1:3 relative ratio, with an overall yield of 30%. (hexane/Et_2_O, 95:5); IR (film) ν_max_: 3075, 2954, 2925, 2853, 1602, 1586, 1488, 1456, 1435, 1263, 1239, 1109, 1045, 1017, 917, 793, 754, 698. ^1^H NMR (CDCl_3_, 300 MHz) *(signals of cis isomer)* δ 5.74 (1H, ddd, *J*=7.4, *J*=10.4Hz, *J*=17.3 Hz, H-1’’), 5.24 (1H, d, *J*=3.5 Hz, H-2), *(signals of trans isomer)*: 5.62 (1H, ddd, *J*=6.9, *J*=10.4, *J*=17.3 Hz, H-1’’), 4.76 (1H, d, *J*=8.7 Hz, H-2), *(signals of both)*: δ  7.14 (3H, m, H-5’, H-5, H-7), 6.94 (5H, m, H-6, H-8, H-2’, H-6’, H-4’), 5.04 (2H, m, H-2’’), 3.83 (3H, s, OMe), 2.92 (2H, m, H-4), 2.83 (1H, m, H-3). ^13^C NMR (CDCl_3_, 75 MHz) δ *(signals of both)*: δ 159.63 (C, C-3’), 152.22 (C, C-8a), 141.46 (C, C-1’), 137.47 (CH, C-1’’), 129.33 (2CH, C-7, C-5’), 127.38 (CH, C-5), 120.60 (C, C-4a), 120.43 (CH, C-6), 119.67 (CH, C-8), 116.52 (CH_2_, C-2’’), 116.29 (CH, C-2’), 113.54 (CH, C-4’), 112.80 (CH, C-6’), 81.91 (CH, C-2), 55.19 (CH_3_, OMe), 42.31 (CH, C-3), 30.76 (CH_2_,C-4). HREIMS (*m/z*) calcd. for C_18_H_18_O_2_ 266.1307 [M]^+^, found 266.1303.

***cis* and *trans* 2-(4-methoxyphenyl)-3-vinyl-2,3-dihydrobenzopyran** **(7d).**

Reaction of benzoxasilocine (**5**) (72 mg, 0.36 mmol) with 4-methoxybenzaldehyde (54 mg, 0.39 mmol) and BF_3_·Et_2_O (91μL, 0.72 mmol) in anhydrous CH_2_Cl_2_ (2 mL) followed by workup as described above yielded **7d** as a *cis/trans* mixture in a 1:5 relative ratio, with an overall yield of 48%. The isomers could be separated by CC (hexane/Et_2_O, 95:5): **7d *cis***: colourless oil; IR (film) ν_max_: 3075, 3038, 3001, 2955, 2927, 2836, 1612, 1584, 1514, 1488, 1456, 1304, 1238, 1175, 1109, 1034, 995, 920, 830, 754. ^1^H NMR (CDCl_3_, 300 MHz) δ 7.28 ( 2H, m, H-2’, H-6’), 7.13 (2H, m, H-5, H-7), 6.90 (4H, m, H-3’, H-6, H-8, H-5’), 5.74 (1H, ddd, *J*=7.4, *J*= 10.4, *J*= 17.3 Hz, H-1’’), 5.23 (1H, d, *J*= 2.6 Hz, H-2), 5.02 (1H, d, *J*=10.7 Hz, H-2’’a), 4.94 (1H, dd, *J*=1.3, *J*=17.2 Hz, H-2’’b), 3.83 (3H, s, OMe), 3.23 (1H, dd, *J*=5.4, *J*=16.0 Hz, H-4a), 2.92 (1H, m, H-3), 2.81 (1H, dd, *J*=4.1, *J*=16.0 Hz, H-4b). ^13^C NMR (CDCl_3_, 75 MHz) δ 158.32 (C, C-4’), 154.18 (C, C-8a), 134.98 (CH, C-1’’), 131.34 (C, C-1’), 129.22 (CH, C-5), 126.94 (CH, C-2’, C-6’), 126.84 (CH, C-7), 120.10 (C, C-4a), 119.97 (CH, C-6), 116.71 (CH_2_, C-2’’), 116.05 (CH, C-8), 112.84 (CH, C-3’, C-5’), 78.65 (CH, C-2), 54.67 (CH_3_, OMe), 41.18 (CH, C-3), 30.26 (CH_2_, C-4). HREIMS (*m/z*) calcd. for C_18_H_18_O_2_ 266.1307 [M]^+^, found 266.1310. **7d *trans***: colourless oil; IR (film) ν_max_: 3073, 3000, 2955, 2925, 2836, 1611, 1513, 1487, 1456, 1302, 1237, 1174, 1033, 994, 919, 829, 753. ^1^H NMR (CDCl_3_, 300 MHz) δ 7.30 (2H, m, H-2’, H-6’), 7.13 (2H, m, H-5, H-7), 6.91 (4H, m, H-3’, H-5’, H-6, H-8), 5.59 (1H, ddd, *J*=3.9, *J*=10.5, *J*=17.4 Hz, H-1’’), 5.03 (1H, dd, *J*= 1.1, *J*= 17.8 Hz, H-2’’b), 5.00 (1H, d, *J*= 8.9 Hz, H-2’’a), 4.75 (1H, d, *J*=8.9 Hz, H-2), 3.84 (3H, s, -OMe), 2.91 (2H, m, H-4), 2.83 (1H, m, H-3). ^13^C NMR (CDCl_3_, 75 MHz) δ 159.44 (C, C-4’), 154.77 (C, C-8a), 137.68 (CH, C-1’’), 132.07 (C, C-1’), 129.29 (CH, C-5), 128.51 (CH, C-2’, C-6’), 127.33 (CH, C-7), 121.32 (C, C-4a), 120.33 (CH, C-6), 116.53 (CH, C-8), 116.21 (CH_2_, C-2’’), 113.77 (CH, C-3’, C-5’), 81.67 (CH, C-2), 55.21 (CH_3_, OMe), 42.36 (CH, C-3), 31.08 (CH_2_, C-4). HREIMS (*m/z*) calcd. for C_18_H_18_O_2_ 266.1307 [M]^+^, found 266.1304.

***cis* and *trans* 2-(2-pivaloyloxyphenyl)-3-vinyl-2,3-dihydrobenzopyran** **(7e).**

Reaction of benzoxasilocine (**5**) (100 mg, 0.5 mmol) with 2-pivaloyloxybenzaldehyde (156 mg, 0.55 mmol) and BF_3_·Et_2_O (126 μL, 1.0 mmol) in anhydrous CH_2_Cl_2_ (3.0 mL) followed by workup as described above yielded **7e** as a *cis/trans* mixture in a 1:3 relative ratio, with an overall yield of 48%. The isomers could be separated by CC (hexane/Et_2_O, 95:5): **7e *cis:*** colourless oil; IR (film) ν_max_: 2954, 2925, 2854, 1751, 1487, 1456, 1272, 1235, 1112, 788, 752, 658, 631. ^1^H NMR (CDCl_3_, 300 MHz) δ 7.61 (1H, dd, *J*=1.7, *J*=7.8 Hz, H-6’), 7.30 (2H, m, H-4’, H-5’), 7.19 (2H, m, H-5, H-7), 7.06 (1H, dd, *J*=3.0, *J*=8.0 Hz, H-3’), 6.93 (2H, m, H-6, H-8), 5.71 (1H, ddd, *J*=8.0, *J*=10.4, *J*=16.6 Hz, H-1’’), 5.34 (1H, d, *J*=1.6 Hz, H-2), 4.97 (1H, dd, *J*=1.8, *J*= 9.8 Hz, H-2’’a), 4.83 (1H, dd, *J*=1.5, *J*= 15.4 Hz, H-2’’b), 3.27 (1H, dd, *J*=10.4, *J*=10.4 Hz, H-4a), 2.84 (2H, m, H-3, H-4b), 1.39 (9H, s, C(CH_3_)_3_). ^13^C NMR (CDCl_3_, 75 MHz) δ 176.44 (C, C=O), 154.76 (C, C-8a), 147.30 (C, C-2’), 134.46 (CH, C-1’’), 131.74 (C, C-1’), 129.83 (CH, C-5), 128.14 (CH, C-4’), 127.95 (CH, C-6’), 127.25 (CH, C-7), 125.29 (CH, C-5’), 121.79 (CH, C-3’), 120.78 (CH, C-6), 120.22 (C, C-4a), 117.74 (CH_2_, C-2’’), 116.74 (CH, C-8), 74.49 (CH, C-2), 40.13 (CH, C-3), 39.12 (C, C(CH_3_)_3_), 31.78 (CH_2_, C-4), 27.12 (CH_3_, (CH_3_)_3_). HREIMS (*m/z*) calcd. for C_22_H_24_O_3_ 336.1725 [M]^+^, found 336.1728. **7e *trans***: colourless oil; IR (film) ν_max_: 3069, 2958, 2926, 2851, 1752, 1585, 1488, 1456, 1396, 1365, 1273, 1241, 1112, 1030, 1014, 920, 771, 752. ^1^H NMR (CDCl_3_, 300 MHz) δ 7.48 (1H, dd, *J*=2.0, *J*=5.6 Hz, H-6’), 7.36 (1H, dt, *J*=1.6, *J*=7.6 Hz, H-4’), 7.27 (1H, m, H-5’), 7.21 (1H, dd, *J*=1.2, *J*=15.3 Hz, H-5), 7.14 (1H, m, H-7), 7.08 (1H, dd, *J*=1.2, *J*= 6.9 Hz, H-3’), 6.91 (2H, m, H-6, H-8), 5.62 (1H, ddd, *J*=6.4, *J*=10.3, *J*=17.1 Hz, H-1’’), 5.04 (3H, m, H-2’’a, H-2’’b, H-2), 2.89 (3H, m, H-3, H-4), 1.32 (9H, s, C(CH_3_)_3_). ^13^C NMR (CDCl_3_, 75 MHz) δ 176.31 (C, C=O), 154.54 (C, C-8a), 148.52 (C, C-2’), 137.10 (CH, C-1’’), 131.93 (C, C-1’), 129.36 (CH, C-5), 128.75 (CH, C-4’), 128.17 (CH, C-6’), 127.45 (CH, C-7), 125.82 (CH, C-5’), 122.44 (CH, C-3’), 121.00( C, C-4a), 120.44 ( CH, C-6), 116.55 (CH, C-8), 116.48 (CH_2_, C-2’’), 76.34 (CH, C-2), 41.62 (CH, C-3), 39.12 (C, C(CH_3_)_3_), 30.56 (CH_2_, C-4), 27.13 (CH_3_, C(CH_3_)_3_). HREIMS (*m/z*) calcd. for C_22_H_24_O_3_ 336.1725 [M]^+^, found 336.1722.

***cis* and *trans* 2-(3-pivaloyloxyphenyl)-3-vinyl-2,3-dihydrobenzopyran** **(7f).**

Reaction of benzoxasilocine (**5**) (64 mg, 0.32 mmol) with 3-pivaloyloxybenzaldehyde (100 mg, 0.35 mmol) and BF_3_·Et_2_O (81 μL, 0.64 mmol) in anhydrous CH_2_Cl_2_ (2.5 mL) followed by workup as described above yielded **7f** as a *cis/trans* mixture in a 1:5 relative ratio, with an overall yield of 42%. (hexane/Et_2_O, 95:5); IR (film) ν_max_: 2951, 2923, 2853, 1756, 1560, 1375, 1260, 1238, 1111, 784, 749, 722. ^1^H NMR (CDCl_3_, 300 MHz) *(signals of cis isomer)* δ 5.73 (1H, ddd, *J*=1.2, *J*=8.6, *J*=12.1 Hz, H-1’’), 5.26 (1H, sa, H-2), *(signals of trans isomer)* δ 5.61 (1H, ddd, *J*=7.1, *J*=10.3, *J*=17.3 Hz, H-1’’), 4.80 (1H, d, *J*=9.5 Hz, H-2), *(signals of both)* δ 7.83 (1H, t, *J*=7.8 Hz, H-5’), 7.21 (1H, m, H-4’), 7.12 (3H, m, H-5, H-7, H-2’), 7.04 (1H, m, H-6’), 6.91 (2H, m, H-6, H-8), 4.95 (1H, d, *J*=16.1 Hz, H-2’’b), 4.91 (1H, d, *J*=12.0 Hz, H-2’’a), 2.92 (2H, m, H-4), 2.80 (1H, m, H-3), 1.38 (9H, s, C(CH_3_)_3_). HREIMS (*m/z*) calcd. for C_22_H_24_O_3_ 336.1725 [M]^+^, found 336.1724.

***cis* and *trans* 2-(4-pivaloyloxyphenyl)-3-vinyl-2,3-dihydrobenzopyran** **(7g).**

Reaction of benzoxasilocine (**5**) (100 mg, 0.5 mmol) with 4-pivaloyloxybenzaldehyde (156.2 mg) and BF_3_·Et_2_O (126 μL, 1.0 mmol) in anhydrous CH_2_Cl_2_ (4.0 mL) followed by workup as described above yielded **7g** as a *cis/trans* mixture in a 1:3 relative ratio, with an overall yield of 47%. (hexane/Et_2_O, 95:5); IR (film) ν_max_: 3073, 2972, 2927, 2871, 1751, 1641, 1697, 1583, 1507, 1486, 1456, 1275, 1237, 1198, 1165, 1114, 1014, 920, 895, 753. ^1^H NMR (CDCl_3_, 300 MHz) *(signals of cis isomer)* δ 5,72 (1H, ddd, *J*=8.3, *J*=10.5, *J*=17.3 Hz, H-1’’), 5.28 (1H, d, *J*=2.35 Hz, H-2), 4.92 (1H, d, *J*=17.1 Hz, H-2’’b), *(signals of trans isomer)* δ 5.60 (1H, ddd, *J*=7.3, *J*=10.3, *J*=17.3 Hz, H-1’’), 5.04 (2H, m, H-2’’), 4.81 (1H, d, *J*=8.9 Hz, H-2) *(signals of both)* δ 7.39 (2H, d,  *J*=7.1 Hz, H-2’,H-6’), 7.10 (4H, m, H-5, H-7, H-3’, H-5’), 6.91 (2H, m, H-6, H-8), 2.92 (2H, m, H-4), 2.80 (1H, m, H-3), 1.38 (9H, s, C(CH_3_)_3_). HREIMS (*m/z*) calcd. for C_22_H_24_O_3_ 336.1725 [M]^+^, found 336.1721.

***Trans-*2-(2-pivaloyloxyphenyl)-3-formyl-2,3-dihydrobenzopyran (10)**

Compound **7e** ***trans*** (22 mg, 0.06 mmol) was dissolved in THF/H_2_O (2 mL, 1:1 v/v) and cooled at 0ºC. The flask was protected from the light. OsO_4_ (44 μL, 0.012 mmol, 2.5% in isopropanol) and NaIO_4_ (50 mg, 0.23 mmol) were added. The mixture was stirred at 0ºC for 5 h, then water was added and was extracted with Et_2_O. The dried (Na_2_SO_4_) extract was concentrated *in vacuo* and purified by chromatography over silica gel, eluting with hexane/ Et_2_O 85:15, to give compound **10** (16mg, 0.047 mmol, 79%) as a colourless oil: ^1^H NMR (CDCl_3_, 300 MHz) δ 9.59 (1H, s, -CHO), 7.50 (1H, dd, *J*=1.6, *J*=7.8 Hz, H-6’), 7.41 (1H, dt, *J*=1.8, *J*=7.6 Hz, H-4’), 7.29 (1H, m, H-5’), 7.15 (3H, m, H-7, H-3’, H-5), 6.95 (2H, m, H-6, H-8), 5.45 (1H, d, *J*=7.2 Hz, H-2), 3.16 (3H, m, H-3, H-4), 1.34 (9H, s, C(CH_3_)_3_). ^13^C NMR (CDCl_3_, 75 MHz) δ 200.49 (C, C=O), 154.12 (C, C-8a), 147.79 (C, C-2’), 130.93 (C, C-1’), 129.62 (CH, C-5), 129.39 (CH, C-4’), 127.97 (CH, C-6’), 127.75 (CH, C-7), 126.25 (CH, C-5’), 122.79 (CH, C-3’), 121.03(CH, C-6), 120.44 (C, C-4a), 116.77 (CH, C-8), 71.87 (CH, C-2), 49.83 (CH, C-3), 39.19 (C, C(CH_3_)_3_), 27.14 (CH_3_, C(CH_3_)_3_), 23.92 (CH_2_, C-4), (C=O signal of OPiv group is missing). HREIMS (*m/z*) calcd. for C_21_H_22_O_4_ 338.1518 [M]^+^, found 338.1521.

*trans* 3-hidroxymethyl-2-(2-hydroxyphenyl)-2,3-dihydrobenzopyran (8).

Compound **10** (26 mg, 0.08 mmol) was dissolved in anhydrous Et_2_O (2 mL), and added to a solution of LiAlH_4_ (9 mg, 0.23 mmol) in EtO_2_ (2 mL). The reaction mixture was stirred at r.t. for 30 minutes. Then, a saturated solution of NH_4_Cl/H_2_O (6 mL) was added, and the solution was extracted with CH_2_Cl_2_. The organic layer was dried over anhyd. Na_2_SO_4_, and the solvent removed *in vacuo*. Flash chromatography of the residue (hexane: Et_2_O 4:6) afforded **8** (15 mg, 0.06 mmol, 76%) as a solid foam: ^1^H NMR (CDCl_3_, 300 MHz) δ 7.50 (1H, dd, *J*=1.6, *J*=7.8 Hz, H-6’), 7.41 (1H, dt, *J*=1.8 Hz, *J*=7.6 Hz, H-4’), 7.29 (1H, m, H-5’), 7.15 (3H, m, H-7, H-3’, H-5), 6.95 (2H, m, H-6, H-8), 5.23 (1H, d, *J*=9.9 Hz, H-2), 3.62 (2H, ddd, *J*=3.4, *J*=12.2, *J*=16.02 Hz, H-1’’), 3.19 (1H, dd, *J*=4.8, *J*=11.5 Hz, H-4), 2.88 (1H, dd, *J*=4.9, *J*=11.4 Hz, H-4), 2.35 (1H, m, H-3). ^13^C NMR (CDCl_3_, 75 MHz) (DEPT) δ 129.71 (CH, C-6’), 129.51 (CH, C-5), 127.82 (CH, C-4’), 127.29 (CH, C-7), 121.13 (CH, C-6), 121.03 (CH, C-5’), 117.16 (CH, C-8), 116.77 (CH, C-3’), 77.06 (CH, C-2), 63.36 (CH_2_, C-1’’), 39.50 (CH, C-3), 27.98 (CH_2_, C-4). HREIMS (*m/z*) calcd. for C_16_H_16_O_3_ 256.1099 [M]^+^, found 256.1095.

***trans*-Homopterocarpan**

(*trans***-**6a,12a-dihydro- 6H,7H-[1]benzopyrano[4,3-*b*][1]benzopyran)

To a solution of **8** (10 mg, 0.03 mmol) in anhyd. THF (1 mL), PPh_3_ (18 mg, 0.07 mmol) and DIAD (14 mg, 0.07 mmol), were added. After 30 min., the solvent was removed *in vacuo.* Flash chromatography (hexane:Et_2_O 85:15) of the residue afforded **9** (4 mg, 0.016 mmol, 56%): ^1^H NMR (CDCl_3_, 500 MHz) δ 7.69 (1H, td, *J*= 7.7, *J*= 1.3 Hz, H-1), 7.25 ( 1H, tt, *J*= 7.7*, J*= 0.84 Hz, H-3), 7.19 ( 1H, t, *J*= 8.1 Hz, H-10), 7.04 (1H, d, *J*= 7.5 Hz, H-8), 7.02 (2H, m, H-11, H-4), 6.95 (1H, dt, *J*= 1.2, *J*= 7.4 Hz, H-9), 6.88 (1H, dd, *J*= 0.9 Hz, *J*= 8.2 Hz, H-2), 4.90 (1H, d, *J*= 10.3 Hz, H-12a), 4.47 (1H, dd*, J*= 3.8 Hz, *J*= 11.2 Hz, H-6a), 3.98 (1H, t, *J*= 11.2 Hz, H-6), 2.89 (1H, dd, *J*= 3.0 Hz, *J*=10.7 Hz, H-7’), 2.68 (1H, dd, *J*= 3.48, *J*= 12.2 Hz, H-7), 2.48 (1H, m, H-6a).^13^C NMR (CDCl_3_, 125 MHz) 154.93 (C, C-11a), 154.17 (C, C-4a), 129.91 (CH, C-8), 129.20 (CH, C-3), 127.61 (CH, C-10), 126.58 (CH, C-1), 121.89 (C, C-12b), 120.96 (CH, C-9), 120.74 (CH, C-4), 120.74 (C, C-7a), 117.36 (CH, C-11), 116.42 (CH, C-2), 73.25 (CH, C-12a), 68.27 (CH_2_, C-6), 32.51 (CH, C-6a), 27.63 (CH_2_, C-7). HREIMS (*m/z*) calcd. for C_16_H_14_O_2_ 238.0994 [M]^+^, found 238.0998.

^19^F NMR

1. Casey, M.; Leonard, J.; Lygo, B.; Procter, G. “Advanced Practical Organic Chemistry” Chapman and Hall, New York, 1990. [↑](#footnote-ref-2)
